# Assessment of Alcohol Consumption and Anxiety as Predictors of Risk of Anorexia and Bulimia in Non-Clinicals Samples

**DOI:** 10.3390/ijerph17176293

**Published:** 2020-08-29

**Authors:** Gisela Pineda-García, Estefanía Ochoa-Ruiz, Gilda Gómez-Peresmitré, Silvia Platas-Acevedo

**Affiliations:** 1 Faculty of Medicine and Psychology, Autonomous University of Baja California, Cal. University 14418, Tijuana International Industrial Park, Tijuana 22390, B.C., Mexico; estefania.ochoa@uabc.edu.mx; 2 Faculty of Psychology, National Autonomous University of Mexico, Av. Universidad 3004, Col. Copilco Universidad, Del. Coyoacán, Mexico City 04515, Mexico; gildag@unam.mx (G.G.-P.); romsip@unam.mx (S.P.-A.)

**Keywords:** anorexia risk, bulimia risk, alcohol, anxiety, drunkorexia

## Abstract

The objective of this study is to assess the effect of alcohol consumption, anxiety, and food restriction before and after consuming alcohol and body image on the risk of anorexia and bulimia in college students from Tijuana, Baja California, through predictive statistical models. A quantitative, descriptive, and cross-sectional design and a non-probabilistic sample of 526 college students from Tijuana, Baja California, México were used. Application of the scales (with acceptable psychometric properties) was conducted in classrooms. Through path analyses, four models were found with adequate indicators of goodness of fit: (1) risk of anorexia in women [Chi Square (X^2^) = 5.34, *p* = 0.376, Adjusted Determination Coefficient (R^2^)= 0.250]; (2) anorexia risk for men (X^2^ = 13.067, *p* = 0.192, R^2^ = 0.058); (3) risk of bulimia in women (X^2^ = 3.358, *p* = 0.645, R^2^ = 0.202); and bulimia risk for men (X^2^ = 14.256, *p* = 0.075, R^2^ = 0.284). The findings provide empirical evidence for the food and alcohol disturbance model.

## 1. Introduction

Alcohol and drug abuse is a worldwide public health concern. The 2018 World Health Organization’s (WHO’s) Global Status Report on Alcohol and Health determined that in 2016, the harmful use of alcohol caused approximately 3 million deaths (or 5.3% of all deaths) [1]. In 2016, an estimated 2.3 billion people were current drinkers and 283 million people aged 15+ years had an alcohol abuse disorder (5.1% of adults) [2]. Mexico has a prevalence of lifetime alcohol use and lifetime DMS IV alcohol dependence of 85.7% and 3.8%, respectively, which are higher than the average prevalence values observed in all countries combined (80.0% and 2.3%, respectively) [3]. According to the National Survey of Drugs, Alcohol and Tabaco Use of 2016 (ENCODAT 2016, by its acronym in Spanish), the excessive consumption of alcohol in the population of 12- to 65-year-olds has increased by 34% since 2011. It is noticeable that, in this report, a higher prevalence of alcohol abuse was found in men [4]. 

With respect to eating disorders, the worldwide epidemiology shows a higher prevalence in women of high-income countries; however, there is a tendency of a growing prevalence in middle- and low-income countries [5]. Latin America has a prevalence of 0.08% in the case of anorexia and 1.61% for bulimia [6]. Nevertheless, in Mexico, the prevalence may be higher, according to the National Survey of Health and Nutrition (ENSANUT) of 2102, in which it was found that 1.3% of the population had a risk of suffering from eating disorders—1.9% in women and 0.8% in men [7].

The concurrent presence of eating disorders and alcohol use has been reported by some authors [8]. Recently, the term “Drunkorexia” has been used to refer to the pattern that involves compensatory conduct after alcohol intake, such as food restriction, excessive exercising, and purges, in order to avoid gaining weight, as well as to increase the intoxication effect of alcohol [9]. 

The word “Drunkorexia” was used for the first time by Kershaw in 2008, to refer to the dual phenomenon of an eating disorder and alcohol abuse [10]. Nowadays, the term proposed is the model of food and alcohol disturbance (FAD). This paradigm points out, in a general way, that (a) compensatory behavior appears before, during, and after drinking alcohol, and (b) there are differences in the manifestation of FAD, depending on sex. For instance, women tend to avoid ingesting food before alcohol consumption, while men exhibit a wider variety of behaviors, such as stopping eating to increase the intoxicative effect of alcohol and being more willing to increase the amount of food consumed in order to prevent a hangover [11].

In a particular way, FAD is based on the transdiagnostic model of eating disorders produced by Fairburn, where this paradigm points out that an overvaluation of the body figure and weight leads to energy restriction prior to excessive alcohol consumption or to help with preventing potential “binge alcohol consumption”. Alternatively, alcohol consumption is conducted without prior food restriction, which may cause a negative effect (guilt, anxiety, and sadness) and this, in turn, produces compensatory behaviors (purges and exercising, among others) and/or food restriction [12] in the transdiagnostic model applied to FAD, and this negative effect can lead to excessive alcohol consumption. In the same way, anxiety is associated with the risk of both anorexia and bulimia [13].

Although the FAD paradigm does not consider the role of body image (BI), it does identify an overvaluation of the weight to form the self-concept. Neither body dissatisfaction nor body distortion have been evaluated as predictors of the alteration of food and alcohol. However, there is sufficient theoretical and empirical evidence that highlights the importance of changes in BI as risk factors and/or characteristics of eating disorders [14,15,16,17].

The evidence presented above shows that there is international empirical research which supports the relationship between alcohol abuse and eating disorders. However, in Mexico, the research on FAD, or drunkorexia, is practically null [18]. Based on the foregoing, the present research’s main objective is to assess the effect of alcohol consumption, anxiety, and food restriction before and after consuming alcohol and body image on the risk of anorexia and bulimia in college students from Tijuana, Baja California, through predictive statistical models. The specific objectives are to describe: (1) the level of alcohol dependence and (2) the degrees of satisfaction/dissatisfaction with body image and its distortion in the participants of the sample.

## 2. Materials and Methods

### 2.1. Design and Participants

The design of this study was quantitative, descriptive, and cross-sectional, and it included a non-probabilistic sample selection. A total of 526 university students from the city of Tijuana, Baja California were included in the study. The average age of those included in the sample was 20.4 years (SD = 2.3). The sample was subdivided into n_1_ = 261 men and n_2_ = 265 women. The participants had different careers but were all from the same university campus: arts, law, economics, languages, engineering, medicine, dentistry, psychology, and tourism. Students between 18 and 30 years old were included, and participants who did not respond to any of the measurement scales were excluded, therefore the response rate of the participants included in the sample was 100%, an indispensable condition for modeling with AMOS; before data deletion, the response rate was 91%. The study was evaluated and accepted by the Bioethics Committee of the Faculty of Medicine and Psychology of the Autonomous University of Baja California (FMP-PI-598/2017-1). All participants agreed to participate in the study and signed the informed consent form.

### 2.2. Instruments

To ascertain the risk of anorexia and bulimia, the Scale of Risk Factors Associated with Eating Disorders (EFRATA) was used [19]. It consists of 54 Likert-type items, whose response options range from “never” = 1 to “always” = 5 (a higher score implies a greater problem or risk). For the purposes of this study, two indices were constructed with the averages of the responses: one of them grouped the behaviors associated with anorexia risk (alpha = 0.816) and the other grouped those related to bulimia risk (alpha = 0.841). The factor analysis yielded a solution that explained 62% of the variance.

Alcohol consumption was measured with the Alcohol Dependency Scale (BEDA), which was designed to measure the dependence of the adult population that consumes alcohol [20]. The scale consists of 15 items and had a reliability level of 0.772 (with the sample data). The response options are never = 0, sometimes = 1, frequently = 2, and almost always = 3. The cut-off scores employed to identify the risk of alcohol dependency were the following: 1 to 10 = low dependence, 11 to 20 = medium dependence, and 21 points or more = severe dependence. The solution obtained with the factor analysis explained 46% of the variance.

To identify calorie restriction before or after alcohol consumption, two Likert questions were designed—“I restrict food consumption before drinking alcohol (RFCB_ALH)/I restrict food consumption after drinking alcohol (RFCA_ALH)”—with five response options (1 = never, 2 = sometimes, 3 = frequently, 4 = very frequently, and 5 = always).

The anxiety variable was measured with the inventory prepared by Beck and Steer in 1993, with 21 Likert-type items. This inventory includes four response options ranging from nothing = 0, to 3 = intensely; a score of 0 to 7 implies “normal” anxiety, 8 to 15 “mild” anxiety, 16 to 25 “moderate” anxiety, and 26 to 63 “severe” anxiety. This scale applied to the study sample obtained an internal consistency of 0.81, while the factor analysis showed a solution that explained 36% of the variance [21].

The level of dissatisfaction or satisfaction with body image was measured from the difference between the value given to the perceived figure minus the value of the ideal figure. A difference equal to zero indicates satisfaction, a positive difference represents dissatisfaction due to the desire to be thinner, and a negative difference indicates the desire to be thicker. The bigger the difference, the greater the degree of dissatisfaction. The perceived and ideal figures were measured with the group of silhouettes of Kearney, Kearney, and Gibney [22], which covers a continuum of weight, from an undernourished or emaciated figure to one with obesity, passing through a normal weight silhouette [23]. The participants were presented with the group of silhouettes at two different moments: at one of the moments, the phrase “Look at the following silhouettes and choose the one that looks more like your body” was used, and at the other, “Look at the following silhouettes and choose the one you would most like to have” was applied.

In the present investigation, the degree of distortion of body image was evaluated through the difference between the perceived figure (grouped into five weight categories) minus the BMI (also in five categories: 1 = extreme thinness, 2 = low weight, 3 = normal weight, 4 = overweight, and 5 = obesity). The cut-off points proposed by WHO were used [24]. A difference equal to zero indicates no distortion, while a positive difference indicates overestimation, and a negative difference indicates underestimation. Distortion levels can range from one to four, and the highest value implies a greater alteration of body image [23].

### 2.3. Procedure

The instruments were collectively applied in classrooms, in a single session, by personnel previously trained, under the supervision of the person responsible for the study. The ethical recommendations of the Mexican Psychological Society for non-intrusive research procedures were followed [25]. Informed consent was obtained.

In data analysis, descriptive and inferential statistics were used, and the chi-square value was specified for comparisons with percentage distributions using the IBM SPSS software for Windows version 22. The path analyses were designed with support from the AMOS module for SPSS. In this sense, it should be noted that the complete models on the risk of anorexia and bulimia were developed with the same predictive variables: body image alteration, body dissatisfaction, anxiety, alcohol dependence, food restriction before consuming alcohol, and food restriction after consuming alcohol. In the Results section, the four solutions that present the best indicators of goodness of fit are presented [26].

## 3. Results

### 3.1. Alcohol Dependence

The results presented in Table 1 show high levels of alcohol dependence (68%), and the highest percentages are located in a low category (58%); only 1.5% of participants reported a high dependence. The differences by sex were not statistically significant (X^2^(3) = 7.08, *p* = 0.069). 

### 3.2. Anxiety

The highest number of participants fell into the “normal” category; however, among the female participants, 23% were identified with “moderate” anxiety and 13% were identified with “severe” anxiety, compared to 14% and 6% in the same categories, respectively, in the male participants. The differences were statistically significant by sex (X^2^(3) = 18.02, *p* = 0.000).

### 3.3. Body Image

In relation to dissatisfaction with body image, it was found that 25% of both men and women were satisfied with their bodies. Of the remaining 75%, a high proportion (62%) of women were dissatisfied with their desire to be thinner. Among men, the highest percentage (42%) of dissatisfaction was due to the desire to be thicker, whilst a smaller percentage wanted to be thinner (33%). The differences by sex were statistically significant (X^2^ = 60.34, *p* = 0.000). In relation to distortion of body image, it was found that 30% of men and 33% of women did not distort their body. The highest percentage of both groups underestimated (63% of men and 60% of women), and this classification of alteration did not show significant differences by sex (X^2^ = 0.609, *p* = 0.737). The figures indicate that a high percentage of women had a low level of underestimation (45% in grade 1) and men had a higher degree of underestimation in level 2 (25% vs. 13% of women at the same level). The degree of distortion did show significant differences from this variable (X^2^ = 22.418, *p* = 0.001)

### 3.4. Anorexia Risk Model for Men

**Figure 1:**
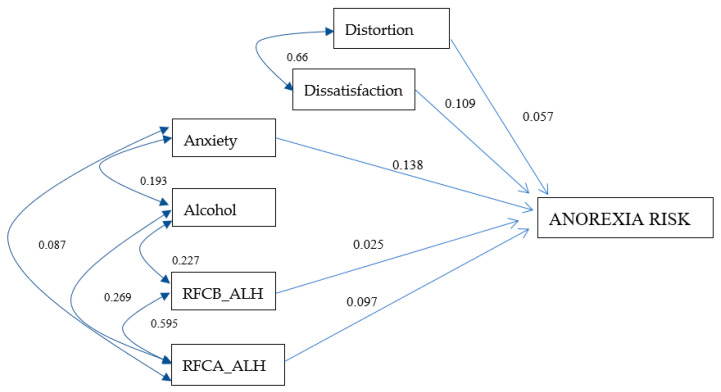
Male anorexia risk model. The values on one-headed arrows are the standardized regression weights and those on each two-headed arrow are the correlations for the model. The shapes in the diagram are defined as follows: small ovals are measurement or prediction errors; rectangles, measured variables. RFCB_ALH: food restriction before consuming alcohol. RFCA_ALH: food restriction after consuming alcohol. X^2^ = 13.067, *p* = 0.192, CFI = 0.989, PNFI = 0.457, RMSEA = 0.037, and R^2^ = 0.058.

A model with good levels of goodness of fit was obtained [X^2^ = 13.06, *p* = 0.192, Root Mean Square Error of Approximation (RMSEA) = 0.037], with a medium degree of parsimony [Parsimonious Normed Fit Index (PNFI) = 0.457] and a 6% prediction variance of anorexia risk in the sample of men (R^2^ = 0.058). In Figure 1, the correlation values are presented, as well as the standardized effects of the independent variables on the dependent variables. In this figure, it is highlighted that for each unit that increases the alteration, dissatisfaction, anxiety, RFCB_ALH, and RFCA_ALH, a low increase in the risk of anorexia occurs, with values of 0.057, 0.109, 0.138, 0.025, and 0.097, respectively.

### 3.5. Anorexia Risk Model for Women

**Figure 2:**
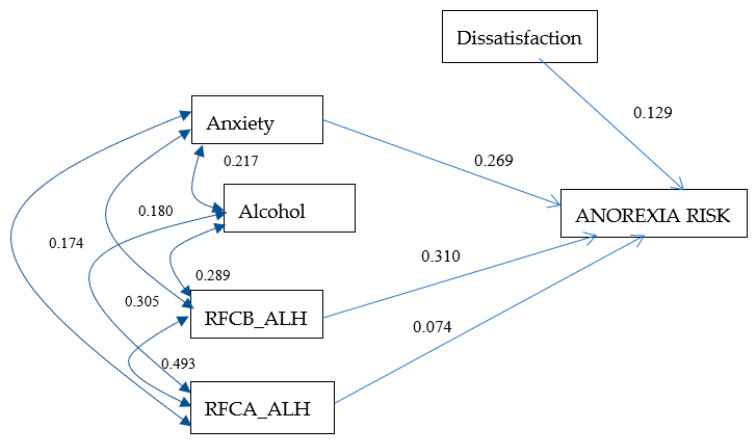
Anorexia risk model for women. The values on one-headed arrows are the standardized regression weights and those on each two-headed arrow are the correlations for the model. The shapes in the diagram are defined as follows: small ovals are measurement or prediction errors; rectangles, measured variables. RFCB_ALH: food restriction before consuming alcohol. RFCA_ALH: food restriction after consuming alcohol. X^2^ = 5.34, *p* = 0.376, CFI = 0.998, PNFI = 0.325, RMSEA = 0.016, and R^2^ = 0.250.

The model of anorexia risk in women resulted in good indicators of goodness of fit (X^2^ = 5.34, *p* = 0.376, RMSEA = 0.016), with 25% of the variance explaining the risk of anorexia. Figure 2 includes, among others, the correlations and the values of the effects of the predictor variables on the main dependent variable. It highlights the size of the effect of dissatisfaction (0.129), anxiety (0.269), food restriction before consuming alcohol (0.310), and RADC_ALH (0.074) on the risk of anorexia. Among them, it is notable that, for each unit that increases RFCB _ALH, the risk of anorexia increases by 0.31.

### 3.6. Bulimia Risk Model for Men

**Figure 3:**
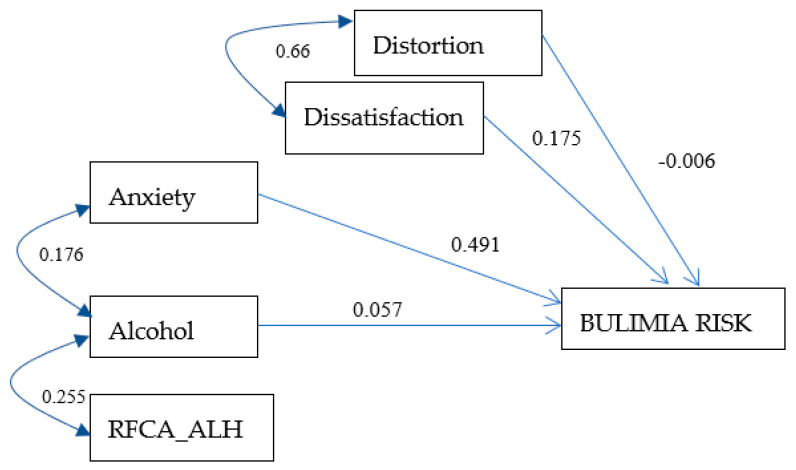
Bulimia risk model for men. The values on one-headed arrows are the standardized regression weights and those on each two-headed arrow are the correlations for the model. The shapes in the diagram are defined as follows: small ovals are measurement or prediction errors; rectangles, measured variables. RFCA_ALH: food restriction after consuming alcohol. X^2^ = 14.256, *p* = 0.075, CFI = 0.977, PNFI = 0.507, RMSEA = 0.055, and R^2^ = 0.284.

A model of the risk of bulimia was developed for the sample of men, with adequate values of goodness of fit (X^2^ = 14.25, *p* = 0.075, RMSEA = 0.055), a medium level of parsimony (PNFI = 0.507), and 28% of the variance being explained. In Figure 3, the effects of the variables distortion (−0.006), dissatisfaction (0.175), anxiety (0.491), and alcohol dependence level (0.057) on the risk of bulimia are visualized. This figure also shows the correlation rates for the predictor variables.

### 3.7. Bulimia Risk Model for Women

**Figure 4:**
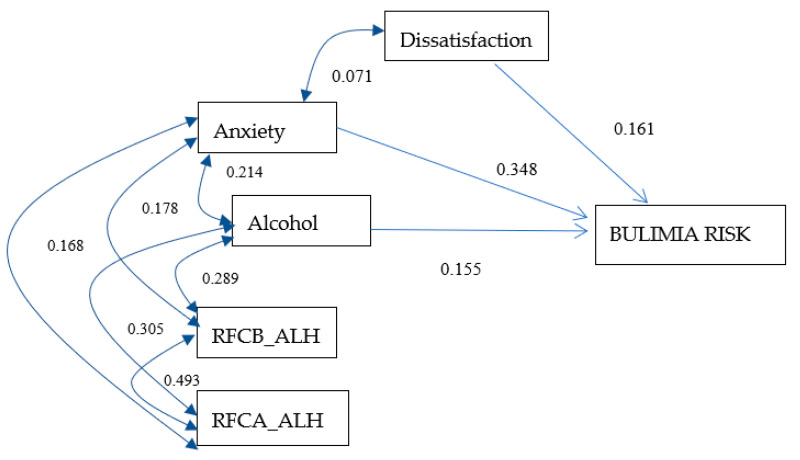
Bulimia risk model for women. The values on one-headed arrows are the standardized regression weights and those on each two-headed arrow are the correlations for the model. The shapes in the diagram are defined as follows: small ovals are measurement or prediction errors; rectangles, measured variables. RFCB_ALH: food restriction before consuming alcohol. RFCA_ALH: food restriction after consuming alcohol. *X*^2^ = 3.358, *p* = 0.645, CFI = 1.0, PNFI = 0.327, RMSEA = 0.000, and R^2^ = 0.202.

Figure 4 includes the correlation indexes for the predictor variables, as well as the standardized regression values of dissatisfaction (0.161), anxiety (0.348), and level of alcohol dependence (0.155) for the risk of bulimia in the sample of women. This model resulted in adequate indicators of goodness of fit (X^2^ = 3.35, *p* = 0.645, RMSEA = 0.000), a low level of parsimony (0.327), and an explained variance percentage of 20% for the risk of bulimia in women.

## 4. Discussion

The first specific objective of the present investigation was to ascertain the level of dependence on alcohol consumption in the sample under study, and the findings indicate that the majority of students have low dependence levels (57.6%). Although the differences by sex were not significant, greater cases of a medium and high dependence were identified in men than in women, coinciding with the ENCODAT (2017). Even though the prevalence of alcohol dependence found in this sample of university students was low, it is still relevant, since a young Mexican population is more prone to increase their alcohol consumption, as indicated by the ENCODAT, with an excessive increase in alcohol intake in 2017, compared to its data in 2011 [4].

The second specific objective was to determine the degree of distortion and dissatisfaction with body image. The findings for both variables were not particularly surprising, since they are in accordance with the previous reports, in which high levels of dissatisfaction with the desire to be thinner in female participants and the desire to be thicker in the sample of men were found, reinforcing the evidence that the ideal of beauty is thinness among women and thickness of muscle among men [27]. Similar to results found in other Mexican samples, high levels of body underestimation were found, especially in men [27,28]. These results are of special interest since both distortion and body dissatisfaction can threaten people’s health. On one hand, dissatisfaction and overestimation are widely described in cases of anorexia, and on the other hand, body underestimation is associated with an increased risk of developing and maintaining obesity [15,16,17].

Regarding the main objective, in each of the models, the relevant role of anxiety in predicting both the risk of anorexia and bulimia is highlighted, being the variable with the greatest effect on them, with the exception of the risk model for anorexia in women, in which food restriction before consuming alcoholic beverages had a greater influence on the dependent variable. This result is especially interesting because it coincides with the proposal of the food and alcohol disturbance model and the definition of drunkorexia [8,10]. As mentioned, the term drunkorexia is used to identify, among others, food restriction before consuming alcohol, with the purpose of avoiding weight gain. The food and alcohol disturbance paradigm points to the same relationship between variables, emphasizing that it is more characteristic in women [6]. Likewise, a risk group for drunkorexia is university women, because they are more prone than their male counterparts to limit their food intake before consuming alcohol [29,30,31] and also face greater pressure to be thin [32,33,34]. This situation can be aggravated since, in addition, university students in general are pressured to consume excess alcohol [35,36,37]. On the other hand, the risk model of anorexia for men had a low prediction of food restriction before and after consuming alcohol. In the case of this model, anxiety and alcohol consumption had a greater effect on the dependent variable, which is a finding in itself. It also supports the FAD paradigm, which identifies variations in the relationship between food and alcohol consumption in the group of men [11].

Continuing with the main objective, in the case of bulimia models (for women and men), it is important to mention that anxiety and alcohol consumption had a greater influence. Caloric restriction before and after consuming alcohol had no direct effect on the risk of bulimia. This finding was expected, since the literature reports, to a lesser extent, an association between food restrictions before consuming alcohol and bulimic behavior. As mentioned, this type of restriction is more frequent in women with a predisposition to anorexia. The literature frequently reports an association between alcohol consumption and eating disorders, especially bulimic behaviors [38,39,40].

The relevance of anxiety as a predictive variable of the risk of both eating disorders has been confirmed once again with the findings of the present study. In this study, anxiety predicted the behavior of bulimia and also that of anorexia in the sample of men and women [13,15,28,41]. The role of anxiety in the explanation of anorexia has been highlighted by various authors, who have pointed out that excessive concern about body image produces anxiety, which is a situation that leads people to engage in restrictive eating behaviors [15]. In the case of bulimic behavior, it can be generated by a failure to control the negative effects, such as anxiety [42]. Therefore, eating food in excess would be used as a kind of negative coping strategy to reduce anxiety. However, this hypothesis was not tested in the present investigation, so it is pertinent to carry out other studies that prove or refute this hypothesis.

The main limitation of the investigation is the non-randomized selection of the sample, which has the consequence that the results are not generalizable to the population of origin. It is therefore suggested that, in future research, random samples are used. Another limitation was the use of self-reporting instruments, as it suggests the use of structured interviews and the design of longitudinal research. Finally, based on the results, the research identified important implications for the health of young people. The high figures of alcohol dependence highlight the importance of the implementation of strategies that seek to prevent and/or correct alcoholism, and such interventions may be based on the theories of self-efficacy [43] and the process model of action in health [44] in order to promote healthy behaviors in the youth population. That anxiety and body image problems are predictors of the risk of anorexia and bulimia shows the necessity of the design and evaluation of programs that seek to reduce anxiety states and traits, as well as to improve the body image of people in order to prevent the appearance of eating disorders, and this can be achieved through interventions that are based on the cognitive theory of mindfulness [45] and theory of cognitive dissonance [46] that have proven their empirical efficacy [47].

## 5. Conclusions

Considering the impossibility of generalization, the main conclusions of the research are as follows: (a) four specific models were obtained for men and women that explain the risk of anorexia and bulimia with adequate values of goodness of fit; (b) the risk of anorexia in women was 25%, and food restriction before alcohol consumption and anxiety were the variables with the greatest influence on said risk, providing evidence for the FAD model; and (c) anxiety and dissatisfaction with body image were the only constant variables with a direct influence on the risk of anorexia and bulimia, with the first one having the greatest effect size in all models, with the exception of the risk of anorexia in women.

## Figures and Tables

**Table 1:** Percent distribution of alcohol dependence level by sex.

Sex			Total
Men		Women	
No dependency	27.2%	36.6%	31.9%
Low dependence	60.5%	54.7%	57.6%
Medium dependency	10.0%	7.9%	8.9%
High dependence	2.3%	0.8%	1.5%
Total	100.0%	100.0%	100.0%
